# Audiovisual Integration Enhances Customer Perception of Artisanal Bread Sounds

**DOI:** 10.3390/foods14213714

**Published:** 2025-10-30

**Authors:** Tianyi Zhang, Maciej Chmara, Charles Spence

**Affiliations:** 1 Crossmodal Research Laboratory, Department of Experimental Psychology, University of Oxford, Oxford OX1 3PS, UK; 2 Institute of Product and Process Design, Berlin University of the Arts, Hardenbergstraße 33, 10623 Berlin, Germany

**Keywords:** artisan bread, audiovisual integration, multisensory, food sounds, consumer perception

## Abstract

Auditory cues are an important, though often overlooked, component of the multisensory experience of food consumption, directly influencing consumer perception and enjoyment. This study investigates how prior food-related experiences affect the perception and preference for food sounds, with a focus on artisanal bread, a popular staple food with distinctive auditory characteristics. A group of 113 participants was recruited and assigned to one of the two groups: 53 attended a bread-making workshop to establish enriched audiovisual associations, while 60 watched bread-making videos online, which represented a comparatively limited form of sensory engagement. Participants rated their perceived comfort levels for three distinct bread-related food sounds before and after the intervention. Sound recognition performance was also assessed as well as the appeal of the sounds. The results revealed that those who attended the workshop evaluated the close-up food sounds significantly more positively than those who watched the videos instead. Furthermore, regression analyses revealed that greater visual involvement during the workshop/watching videos was associated with increased comfort and decreased annoyance for the close-up bread sounds. These findings underscore the importance of multisensory integration experiences, particularly audiovisual integration, in shaping consumer responses and preferences for food sounds. To make sure that consumers feel comfortable and even hungry when they listen to food-related audial content, it is beneficial to incorporate familiar food sounds and, where possible, reinforce these with visual or experiential cues. Content that leverages multisensory associations and aligns with listeners’ prior experiences is likely to be more effective in eliciting positive sensory and emotional responses.

## 1. Introduction

“The sound of a knife cutting through the crust or the sight of butter melting into a slice of warm sourdough—these are amongst life’s simple pleasures” [1].

Sound is rarely the first sense that people associate with cooking and eating, but it is a powerful one, especially in terms of shaping people’s culinary experience [2,3]. From the crackle of a freshly baked baguette to the rhythmic stretching and folding of the dough, auditory cues not only signal food quality but can also make food seem fresher and tastier (e.g., as demonstrated by Zampini and Spence’s ‘sonic chip’ study [4]; see also [5,6]). Despite the importance of the auditory cues that are associated with the preparation and consumption of food, there is a lack of empirical insights into how people evaluate these cues beyond the experience of taste. In other words, the previous literature on people’s food experience has investigated the role of auditory cues in enhancing the taste of food [7,8], yet food sounds could be pleasurable in their own right.

The sounds associated with mastication provide useful cues regarding a food’s texture. So, for example, the crunch of a potato chip or the crackling sounds of the crust of a loaf of bread can enhance the perception of food textures people experience when eating. Even when presented in isolation, cooking-related sounds can evoke mental associations of the corresponding food categories or the anticipation of specific food qualities [9,10,11,12]. These sounds are no accident—artisanal techniques, such as the stretch and fold of sourdough or scoring bread crusts, are carefully honed techniques developed by bakers to produce textures and acoustics that help to define culinary authenticity. However, while gastronomy increasingly embraces multisensory design (e.g., sonic seasoning and soundscape matching; [13,14]), the role of prior multisensory experiences, especially audiovisual integration, in shaping the perception and preference for food sounds remains poorly understood, particularly in more artisanal contexts. This is the research gap that the present study seeks to address.

One relevant example that combines the sound and sight of food is ‘ASMR eating’, which one often finds featured in videos known as mukbang. These food videos focus on capturing the audiovisual cues associated with someone consuming food in a highly sensory manner [15,16]. In order to create an enhanced eating setting, usually sounds in ASMR videos are deliberately engineered to trigger tingle sensations (at least in those who happen to be sensitive to such effects) so as to make the content viscerally satisfying to viewers [17,18,19]. Such content begins to blur the line between the enjoyment associated with the actual consumption of food and the sensory pleasure of merely watching someone else eat, demonstrating the significant role of food sounds in crafting an immersive (or even imaginary) food experience.

By themselves, sounds such as the clinking of a dough scraper may lack meaning without the relevant visual or tactile associations or context. However, it is important to note that auditory cues can trigger memories [20] and moderate consumer preferences [7,21], sometimes enhancing and/or even transcending the boundaries that are normally associated with visual perception [22,23,24]. When we hear the sourdough starter, salt, and water being whisked in a bowl, we anticipate the rapid, repeated clink of metal gently hitting the mixing bowl, without necessarily seeing the scene. Such phenomena resonate with the behavioral and neural evidence that there are audiovisual interactions in the formation and retrieval of visual memories [22]. For instance, those diners who are unfamiliar with bread-making may overlook the existence of the crack(l)ing sounds of the crust, while those who have themselves shaped dough by hand might immediately recognize these sounds and associate them with the craftsmanship and freshness that those food sounds signify. This interplay between sounds and visual memories aligns with gastronomy’s emphasis on storytelling and education, where chefs use workshops or open kitchens to deepen patrons’ sensory engagement by creating contextual memories of cooking and dining [25,26]. Yet, empirical research on how hands-on culinary experiences, especially related to audiovisual integration, shape food sound preferences remains sparse in the food science literature.

There is now considerable evidence to show that when people attempt to retrieve visual and auditory information, there are similar patterns of neural activation as when people perceive multisensory stimuli (e.g., in the visual and auditory cortex, respectively [27]; see Soto-Faraco and Spence’s review [28]). Therefore, memories of a scene can benefit from two sources of relevant information: the auditory and visual cues that were presented during the encoding of the scene [29,30]

Given the lack of empirical research assessing the role of audiovisual integration in shaping people’s perception of food sounds, this study addresses the following research question: How does prior audiovisual experience with bread-making (hands-on workshop vs. watching food-making videos online) influence consumer perception and preference for bread-related food sounds? The question was whether the sounds would become imbued with meaning once the participants had taken part in a very hands-on multisensory bread-making and tasting workshop. The hypotheses were as follows: any audiovisual integration that had either been established by attending the bread-making workshop or else simply by watching relevant videos online would positively enhance people’s evaluations of the artisanal sounds associated with (making) bread (H1); and that greater visual involvement during the workshop or video viewing would be associated with increased preferences for bread sounds (H2). Given these aims, this study contributes to the sensory and consumer sciences by clarifying the mechanisms underlying multisensory integration in food sound perception. The findings are relevant to food product development, marketing, and education, offering practical insights into how sound can be leveraged to enhance consumer enjoyment and acceptance of food experiences.

## 2. Methods

A mixed-design approach was used to evaluate how visual associations influence people’s evaluations of bread-making sounds. There were two between-group factors (high visual association condition: participation in a sourdough bread-making workshop; low visual association condition: watching sourdough bread-making workshop videos online) and two within-group factors: time (before vs. post bread-making interventions), and soundtrack (bread chewing vs. cutting vs. stroking sounds). In other words, each participant evaluated all three bread-related soundtracks at two points in time: both before and after participating in the workshop/watching the workshop videos. The participants in the high visual association condition were able to establish audiovisual associations related to bread-making through actively engaging in the bread-making and tasting process which lasted around 2 h per workshop, while those in the online video watching condition were only able to establish a reduced level of visual association.

### 2.1. Participants

A group of 113 participants took part in the study. The sample size was determined using an a priori power analysis conducted in G*Power 3.1 [31], targeting a small effect size (*f* = 0.15) and a statistical power (1 − *β*) of 0.80 with *α* = 0.05. For a statistical test of ANOVA on repeated measures (within–between interaction), a moderate correlation (i.e., 0.5) between participants’ self-reported measures before and after the interventions was assumed. Therefore, a total sample size of 90 was suggested as required for this design, meaning a minimum of 45 participants per group. A total of 53 of the 113 participants were recruited through in-person sourdough-making workshops (there were three workshops in total, each with around 20–25 people signed up, though inevitably there were a number of no-shows on the day) in the Berlin University of the Arts. The sample of respondents (34% men, 47% women, 9% preferred not to indicate) were residents in Berlin, aged 20 to 66 years, with normal or corrected-to-normal vision and hearing. They were recruited via email, social media, and community advertisements. The data from five of the participants were removed due to missing responses (due to arriving late or missing some of the evaluative questions in between the workshop sessions). Therefore, a total of 48 participants from the workshop group were included in the analysis, *M*_age_ = 36.88 years, *SD*_age_ = 12.60 years. The participants showed a mixed profile of prior baking experiences: 14 indicated that this was their first time making bread, 20 had little bread-making experiences, 12 reported that they occasionally made a loaf of bread at home, while 2 of the participants indicated that they often made bread at home.

As for the control group that watched bread-making videos online, the participants were invited to participate in an online study evaluating sourdough-making videos through Prolific (https://www.prolific.com/), a widely used online research platform that ensures responses from verified users. The target population consisted of German residents who were fluent in English, aged 18 years and older, who had normal audition and vision. These criteria were set on Prolific before the study was released for recruitment to automatically filter out those participants who did not meet the criteria. Data were collected from 60 participants (58% men, 40% women, 2% preferred not to indicate, aged 19 to 57 years). However, 7 participants’ data were eliminated due to incomplete responses or technical issues with playing the videos. Therefore, a total of 53 participants from the online viewing group were included in the final analysis (*M*_age_ = 31.19 years, *SD*_age_ = 8.42 years), with 10 people having no previous experience baking, 21 people having a little experience, 12 people occasionally making a loaf at home, and 9 people who reported frequently making bread at home. Overall, the two groups showed a similar profile in terms of their baking experiences.

### 2.2. Materials

In food videos, creators normally emphasize close-up sounds, such as chewing, crunching, and intimate interactions with the surface of the foods, with the aim being to evoke specific sensory responses in their audience [4,32]. The sounds used in the present study were therefore chosen as they featured close-up food sounds, such as chewing and crunching. Three bread-related sounds that represent different phases of how one consumer could interact with the bread were used in the current study: (1) chewing bread sounds, (2) cutting bread with a knife sounds, and (3) the sound of stroking the crust of a freshly baked loaf. The sounds were recorded in a recording studio with 6-channel microphones. To minimize external and internal background noise (e.g., nature sounds, echo), structure-borne sound and airborne sound were isolated stereophonically with two transducers each attached symmetrically to the decoupled glass plate serving as a base and two cardioid microphones pointed at the action above. The recordings were then mixed and processed with Avid Pro Tools. Due to copyright restrictions, the sound files can only be viewed online: https://osf.io/q4n93/overview?view_only=ad07edf162c446d1a935872f9b0bb565, accessed on 5 October 2025. The soundtracks lasted around 1–2 min for each stimulus. This duration was intended to make sure that the processes of chewing, cutting, and stroking were presented completely around five times for each type of sound, and thus the participants could embed themselves in the sounds. This repetition is also to mimic real life cases where food-related sounds would be played repeatedly to consumers, such as in ASMR videos and soundtracks.

The sourdough-making videos were recorded in a similar setup with the actor presenting the procedure of sourdough-making from mixing ingredients to folding the fermented dough, and finally to the presentation of the freshly baked sourdough. The videos did not feature the actor’s facial features at all. Instead, the cameras were set to focus on the motions of the hands. A demo of the videos featuring the full process of sourdough-making can be found at https://youtu.be/EEQ1yn8Tc9c, accessed on 5 October 2025.

### 2.3. Measures and Procedure

The participants in the workshop condition (i.e., the high visual association condition) were informed that they were invited to take part in a 2 h sourdough-making workshop for free, with designated questionnaires to be completed during the course of the workshop. Upon arrival at the workshop, the participants provided their informed consent to take part in the workshop. The participants were then first requested to provide their demographic information (i.e., age and sex) and their prior experience with bread-making on a 5-point scale: 1 = first time making bread, 2 = have little bread-making experiences, 3 = occasionally make loaf at home, 4 = often make bread at home, and 5 = professional baker. The participants were then invited to listen to sourdough-making-related soundtracks before they took part in the sourdough-making workshop. Three different soundtracks were played, and participants were not informed of the exact content of the sound: 1. bread chewing; 2. bread cutting; 3. stroking the crust of bread. The participants were invited to listen to the tracks one-by-one for about 75 s. Each sound was evaluated across four subjective evaluative dimensions, including recognition, perceived comfort, perceived annoyance, and perceived appetitive response on five-point scales (see Table 1). The questions used in the current study were adapted from previous studies on the general impression of perceived pseudo-chewing sounds on the effect of auditory information, on food sound recognition, and on the appetitive responses to food-related sensory cues [33,34,35].

**Figure 1:**
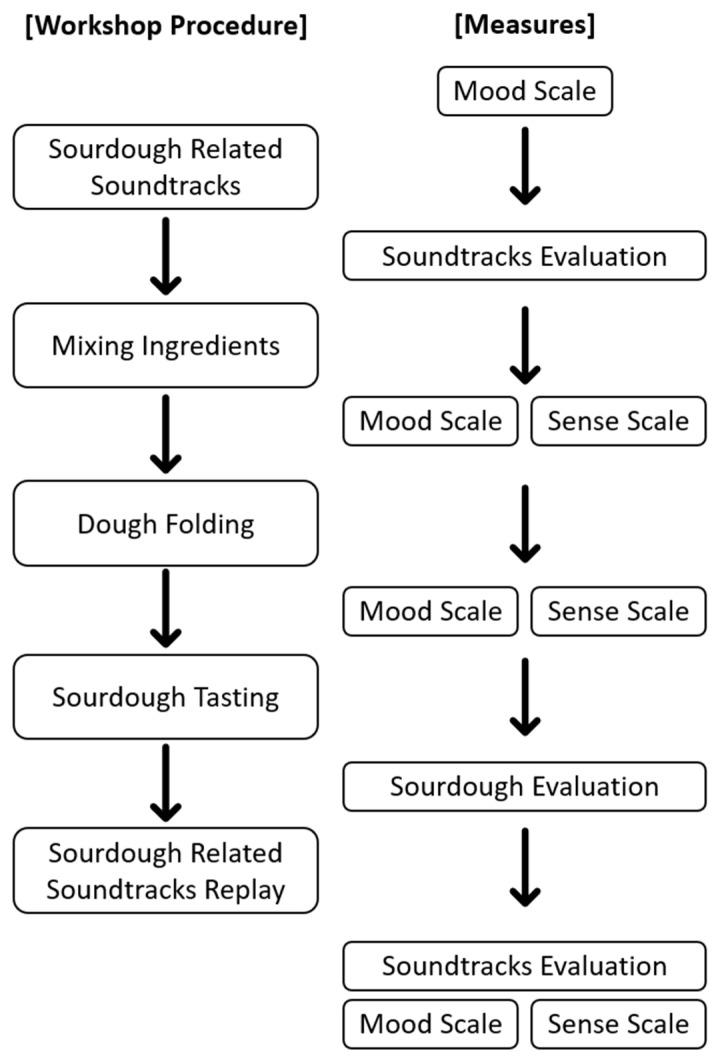
The procedure for the sourdough-making workshop and the sequence of response measures. As shown, the participants were required to fill out the soundtrack evaluations before they mixed the ingredients when they listened to the soundtracks for the first time and after the sourdough tasting when they listened to the soundtracks for the second time.

The soundtrack evaluation was part of a comprehensive well-being study on artisanal activities [36]. Therefore, the workshops were designed purposely to engage multisensory aspects throughout the sourdough-making process. In addition to the soundtrack evaluations that were assessed and presented in the current study, the workshop involved the evaluation of mood states, and the sourdough tasting session (see Figure 1 for an illustration of the workshop procedure and how measures were collected between the sourdough-making phases). In the workshop guided by sourdough baker Maciej Chmara from the Berlin University of the Arts, the participants poured water and active sourdough starter into large bowls. The participants were invited to sniff the aroma of the sourdough starter before adding it to the bowl of ingredients.

**Figure 2:**
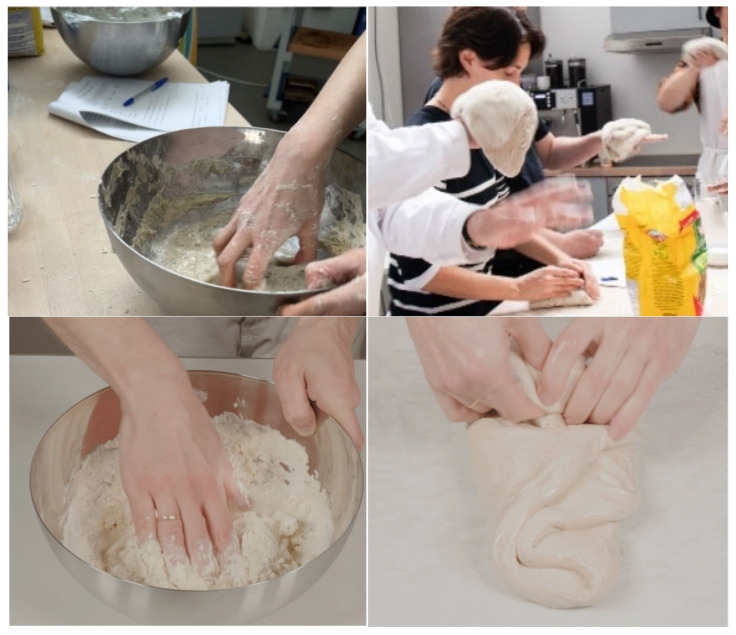
Demonstration of the ingredient mixing procedure (**top-left**), and participants balancing sourdough on the back of their hands as part of the dough folding procedure (**top-right**). For the online group, participants viewed videos of bread making, which were also separated into ingredients mixing (**bottom left**), dough folding (**bottom right**) and bread being made.

The instructor then asked the participants to splash their fingers in the bowl to stir the watery ingredients (see Figure 2). The flour and salt were then added to the mixture and were mixed with the fingers until the ingredients had been fully incorporated. The next step involved stretching and folding pre-fermented dough. This process involved stretching a portion of the dough and then folding it over the rest, rotating the dough and repeating this action until the dough gains strength, taking about 10–15 min. Pre-fermented dough was prepared for the workshop participants in advance. They were invited to stretch the dough and closely perceive its weight on the back of their hand and the movement of it between hands as if they were doing yoga, with the dough being a kind of exercise equipment. After stretching and folding the dough, the participants were provided with several freshly made and pre-made sourdough samples and were invited to complete several tasting and evaluation sessions. After the tasting session, participants listened to the five soundtracks related to bread again and re-evaluated along the four items. Participants were not instructed to pay attention to the specific food sounds (i.e., chewing, cutting, and stroking bread) during the workshop.

The sense scales were intended to assess the intensity of the participants’ sensory experiences associated with each sourdough-making procedure. The participants evaluated the uses of olfactory (e.g., aroma from sourdough ingredients), auditory (e.g., sound of mixing), visual (e.g., sight of sourdough slices), haptic (e.g., folding and balancing the dough on the back of the hands), and gustatory (e.g., tasting the sourdough slices) modalities, acknowledging that flavor clearly engages more senses than merely just gustation.

For the online condition, participants went through the same procedure (as described in Figure 1). The participants were invited to watch video clips of sourdough-making (as shown in Figure 2). Before the videos were presented, to ensure that participants had connected their head(ear)phones and could hear the sound samples at a comfortable listening level, a music sample was played to people followed by the following instruction: “Slowly adjust your device’s volume to a level that you can clearly hear the music and that you feel comfortable with. Once you have completed all the steps above, please keep your volume in this setting during the entire study.” After that, participants were shown videos of the mixing of ingredients, dough folding, and freshly baked bread coming out of the oven and being cut. Each video clip lasted around one to two minutes and participants were not able to skip the videos till they were finished. Just as in the workshop, no instruction was given to the online participants to pay attention to the sounds in the videos specifically.

A full list of the questionnaires used in the study, including the mood scales, sense scales, and soundtrack evaluation scales, can be found in the Appendix A.

### 2.4. Empirical Analysis

The following analyses were carried out: (1) descriptive analyses of sound recognition, perceived comfort, annoyance, and appetite; (2) group comparisons of the intensities of the use of vision as a manipulation check of the visual association levels in the workshop group vs. the online video watching group; (3) analyses of the impact of visual association across evaluative dimensions were tested with repeated measured analysis of covariance (ANCOVA), with visual association levels as the between-group variable and pre-/post- intervention time-points as the within-group variable, and sex (male = 0, female = 1), age, and baking experiences as the covariates; (4) regression analyses exploring the influence of sensory engagement (i.e., intensity of the uses of vision and hearing) on the changes in food sounds perception. Analyses steps 1–3 were conducted with SPSS 29.0 and step 4 was conducted with R 4.4. All statistical analyses were conducted at the confidence level 0.05. Q-Q plots of the variables measured were examined before running the parametric analyses.

## 3. Results

### 3.1. Preliminary Analysis

Table 2 presents the descriptive results depicting the mean and standard deviations of the perceived comfort, levels of recognition and annoyance, and the appetizing effects of the three soundtracks. Before the visual association interventions, the chewing sound was rated as the most easily recognized but the least pleasant or comfortable to listen to, both for the workshop condition and the online condition. Table 3 presents the average ratings (and standard deviations) of how intense vision and hearing were used in each of the sourdough-making procedures. Overall, vision was used intensely both in the workshop and when watching online videos throughout the bread-making process, while hearing was not used in the workshop as much as when watching online videos in the ingredients mixing and dough folding stages. This could be understood as meaning that for in-person workshops more sensory experiences were available from the other senses, e.g., olfaction and touch.

Group comparisons revealed that vision was used significantly more intensely in the workshop as compared to the online condition (*M*_workshop_ = 4.26 vs. *M*_online_ = 3.74, *F* (1, 98) = 13.70, *p* < 0.001). Pairwise comparisons further demonstrated significantly higher ratings of the intensity of the usage of vision in the workshop as compared to online conditions during the mixing of ingredients (mean difference = 0.66, *p* < 0.001) and the folding of the dough (mean difference = 0.80, *p* < 0.001), and during the tasting session (mean difference = 0.11, *p* = 0.538). Overall, the use of vision was more intense for those in the workshop group as compared to those in the online group. Additionally, the time that people spent in the workshop (approximately 2 h) was much greater than participants online (with a median of 16 min). Therefore, the participants in the workshop group were able to establish a higher level of visual associations than those in the online group.

### 3.2. Evaluations of Food Sounds

The recognition, perceived comfort, perceived annoyance, and appetitive responses to the three bread-related sounds were averaged before they were entered into the generalized linear models. The detailed results for each soundtrack (i.e., bread chewing, cutting, and stroking sounds) are presented in Table 4.

**Figure 3:**
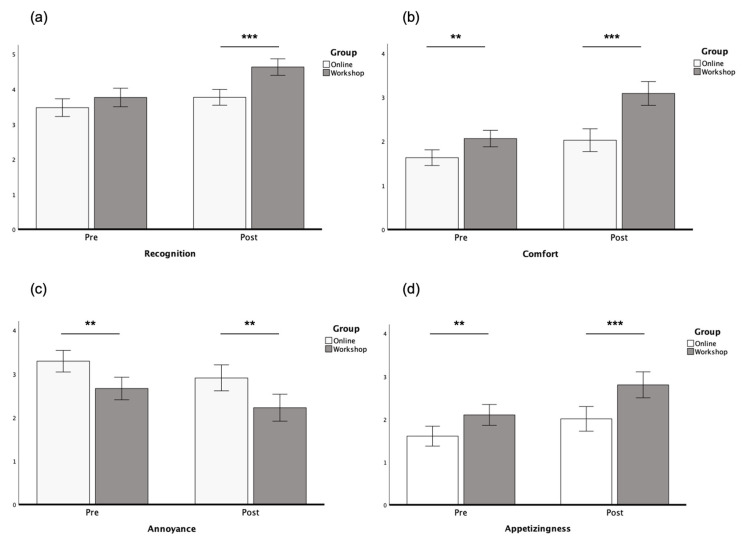
Estimated marginal means of recognition (**a**), perceived comfort (**b**), perceived annoyance (**c**), and appetizingness (**d**) for bread-related sounds before and after the workshops/online video watching (all scaled on 1 to 5). Covariates appearing in the model are evaluated at the following values: experience = 1.70 (scaled on 1 to 5), sex = 0.64 (female = 1, male = 0), age = 33.92 years. Error bars: 95% CI. Note: *** *p* < 0.001. ** *p* < 0.01.

**Recognition** Repeated measured ANCOVA results showed a significant interaction effect of visual association levels (high: workshop vs. low: online) and time (pre- and post- intervention) on the recognition of sounds (*F*(1, 95)  =  10.34, *p*  = 0 .002, η_p_^2^  =  0.098). As shown in Figure 3a, the recognition of bread sounds in the workshop group (pre: *M*_workshop_ = 3.76, *SE*_workshop_ = 0.13; post: *M*_workshop_ = 4.63, *SE*_workshop_ = 0.11) increased significantly more than the online group (pre: *M*_online_ = 3.47, *SE*_online_ = 0.13; post: *M*_online_ = 3.77, *SE*_online_ = 0.11). More specifically, there was a main effect of time (*F*(1, 95)  =  4.73, *p*  = 0 .032, η_p_^2^  =  0.047), indicating a significant increase in the recognition of sounds after taking part in the workshop or watching bread-making videos online. There was also a significant main effect of visual association levels (high: workshop vs. low: online), *F*(1, 95)  =  12.55, *p*  <  0.001, η_p_^2^  =  0.117.

**Perceived Comfort** There was a significant interaction effect of visual association levels (high: workshop vs. low: online) and time (pre- and post- intervention) on the comfort of listening to close-up food sounds (*F*(1, 95)  =   16.78, *p*  < 0 .001, η_p_^2^  =  0.150). As shown in Figure 3b, participants in the workshop group (pre: *M*_workshop_ = 2.06, *SE*_workshop_ = 0.09; post: *M*_workshop_ = 3.08, *SE*_workshop_ = 0.14) rated the sounds as significantly more comfortable to listen to than those in the online group (pre: *M*_online_ = 1.63, *SE*_online_ = 0.09; post: *M*_online_ = 2.02, *SE*_online_ = 0.13). There was a significant main effect of time (*F*(1, 95)  =  11.45, *p*  = 0 .001, η_p_^2^  = 0 .108), indicating an increase in perceived comfort towards the bread-related sounds after having taken part in the workshop or after having watched bread-making videos online. There was also a significant main effect of visual association levels (high: workshop vs. low: online), *F*(1, 95)  =  23.59, *p*  < 0 .001, η_p_^2^  =  0.199.

**Perceived Annoyance** There was no significant interaction effect of visual association levels (high: workshop vs. low: online) and time (pre- and post- intervention) on annoyance sounds (*F*(1, 95)  =  0.11, *p*  = 0 .745, η_p_^2^  = 0 .001). There was also no main effect of time (*F*(1, 95)  =  1.72, *p*  =  0.192, η_p_^2^  =  0.018). However, there was also a significant main effect of visual association levels (high: workshop vs. low: online), *F*(1, 95)  =  11.81, *p*  <  0.001, η_p_^2^  = 0 .111. The perceived annoyance of sounds in both groups decreased to a similar level (pre: *M*_workshop_ = 2.67, *SE*_workshop_ = 0.13; post: *M*_workshop_ = 2.22, *SE*_workshop_ = 0.16) and was significantly lower than the online group (pre: *M*_online_ = 3.29, *SE*_online_ = 0.12; post: *M*_online_ = 2.91, *SE*_online_ = 0.15), as depicted in Figure 3c.

**Appetitive Responses** There was no significant interaction effect of visual association levels (high: workshop vs. low: online) and time (pre- and post- intervention) on appetitive responses to the sounds (*F*(1, 95)  =  2.22, *p*  = 0 .140, η_p_^2^  =  0.023). There was a main effect of time (*F*(1, 95)  = 9.45, *p*  =  0.003, η_p_^2^  =  0.090). There was also a significant main effect of visual association levels (high: workshop vs. low: online), *F*(1, 95)  =  13.37, *p*  < 0 .001, η_p_^2^  =  0.123. The perceived appetizingness of sounds in the workshop group (pre: *M*_workshop_ = 2.10, *SE*_workshop_ = 0.12; post: *M*_workshop_ = 2.80, *SE*_workshop_ = 0.15) and online group (pre: *M*_workshop_ = 1.60, *SE*_workshop_ = 0.12; post: *M*_workshop_ = 2.01, *SE*_workshop_ = 0.14) both increased significantly (see Figure 3d).

Overall, the ANCOVA results supported H1, showing that audiovisual integration experiences positively enhanced people’s evaluations of the relevant food sounds, i.e., in the current study, artisanal sounds associated with (making) bread.

### 3.3. Predicting Sound Perception with Sensory Intensities

A multiple linear regression analysis was conducted to examine the influence of sensory engagement (vision and hearing) on the change in perception of sounds over time, including comfort, annoyance, hunger, and recognition of bread-related sounds. Vision and hearing were entered as predictors. The correlations of the variables are presented in Table 5.

For **comfort**, the overall model was significant, *F*(2,97) = 3.44, *p* = 0.036, *R*^2^ = 0.066. Vision significantly predicted comfort (*β* = 0.22, *p* = 0.026), indicating that higher visual engagement was associated with greater increase in perceived comfort of food sounds. Hearing did not significantly predict comfort (*β* = −0.09, *p* = 0.267), suggesting that auditory engagement did not contribute to increased comfort.

For **annoyance**, the model was significant, *F*(2,97) = 3.48, *p* = 0.034, *R*^2^ = 0.067. Vision significantly predicted annoyance (*β* = −0.26, *p* = 0.018), indicating that higher visual engagement was associated with more decrease in annoyance towards food sounds. Hearing did not significantly predict annoyance (*β* = −0.12, *p* = 0.21).

**Figure 4:**
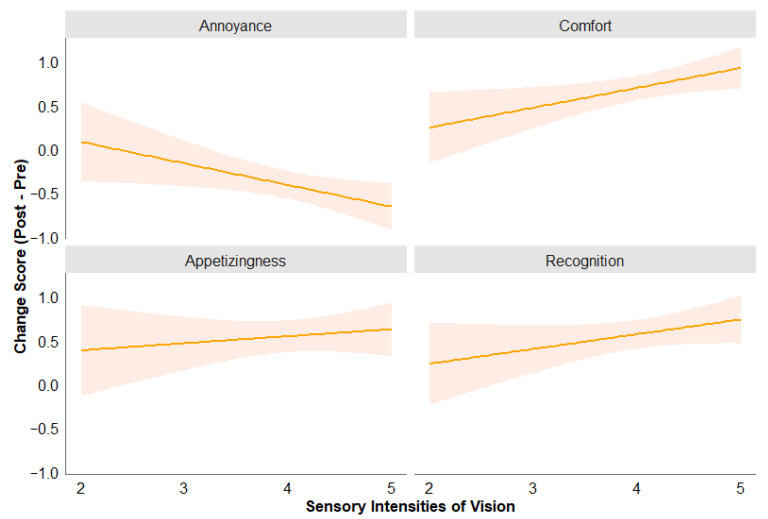
Visual engagement significantly predicted the decrease in annoyance and increase in comfort (**top**), but was not significant for predicting appetizingness and recognition (**bottom**).

For **appetizingness and recognition**, the overall model was not significant: appetizingness, *F*(2,97) = 0.27, *p* = 0.763, *R*^2^ = 0.006; recognition, *F*(2,97) = 2.74, *p* = 0.069, *R*^2^ = 0.054. Figure 4 shows the relationship between visual engagement and the changes in participants’ dependent measures.

The results of the regression analyses therefore partially supported H2 that greater visual involvement during the workshop or video viewing would be associated with increased perceived comfort and decreased annoyance for bread sounds. However, the associations between visual involvement and bread sound recognition and perceived appetizingness were not observed.

## 4. Discussion

### 4.1. Theoretical and Practical Implications

This study demonstrates that audiovisual integration—particularly through immersive, hands-on culinary experiences—significantly enhances the perception and appreciation of food-related sounds. Consumer’s prior sensory experiences directly influence their preferences for food-related sensory cues (i.e., auditory cues). Participants in the multisensory bread-making workshop exhibited improved recognition of bread sounds (chewing, cutting, and stroking), reduced annoyance, and increased appetitive responses compared to those who passively watched videos. In fact, just as asserted by Van Drie [37]: “Sound, like food, is corporeal. Listening, like cooking, is multisensorial” (also in [26]). The findings from the present study underscore that contextual engagement transforms auditory cues into meaningful sensory narratives, highlighting the importance of multisensory integration in shaping food enjoyment and acceptance, a central concern in sensory science and consumer research. The correlation between visual engagement during the workshop and heightened sound preference underscores the role of multisensory memory in shaping gastronomic experiences.

From more of a cognitive perspective, this study’s findings indicate that memory and emotion play a powerful role in sensory evaluation [38]. The multisensory experiences created in bread-making workshops often form richer, more enduring episodic memories. When individuals later encounter similar food sounds, these auditory cues evoke positive workshop experiences, thereby reinforcing favorable attitudes and emotional responses toward both the sounds and the food. This aligns with prior findings that multisensory cues enhance memory encoding and retrieval [39,40], making food experiences more memorable and emotionally resonant.

Importantly, these findings contribute to the growing body of the literature on sensory and consumer science by demonstrating how sensory interventions can be strategically used to enhance consumer comfort and enjoyment of food-related sounds [26,41]. Notably, both workshop and video groups reported reduced annoyance, though the workshop group showed stronger effects. This suggests that even limited audiovisual exposure (e.g., videos) can moderately enhance sound tolerance, while immersive multisensory experiences help to deepen emotional and sensory connections. For example, the workshop’s tactile elements—kneading dough, scoring loaves—likely anchored sounds to positive, embodied memories, mitigating potential aversions. This aligns with prior work linking contextualization to ASMR effectiveness [42] and offers a pathway to address misophonia in dining settings by reframing triggers like chewing as culturally or craft-rooted experiences [43,44].

A key finding to emerge from the present study was the inherent appeal of freshly baked bread crust’s stroking sounds, which participants consistently rated as comfortable and desirable, even pre-intervention. This echoes Poerio et al.’s [45] identification of scratching surfaces as universal ASMR triggers, suggesting that such sounds possess intrinsic acoustic properties (e.g., rhythmic, high-frequency textures) that transcend contextual familiarity. Conversely, chewing sounds—often linked to misophonia—did not trigger aversion here, possibly due to their framing within food-making contexts rather than isolated consumption [18,19,45].

### 4.2. Limitations and Future Research

The multisensory nature of the bread-making workshop, which included not only auditory but also visual, olfactory, and tactile cues, makes it difficult to isolate the specific effects of sound. Additionally, the use of self-report measures may introduce subjective bias, although it should be noted that such approaches are standard practice in sensory science. The sample may not fully represent the broader population, and the focus on bread-related sounds may limit the generalizability of the findings to other foods or cultural settings. Future research could disentangle these factors by controlling individual sensory inputs. Additionally, exploring whether sound preferences correlate with food attributes (e.g., energy density, cultural symbolism) could uncover deeper links between auditory cues and perceived quality [46,47,48]. Cross-cultural comparisons—such as contrasting bread-making sounds with wok-frying or chocolate tempering—may reveal universal versus culture-specific auditory preferences.

For culinary professionals, these findings hint at the potential benefits associated with integrating sound-centric storytelling into workshops, menus, and marketing. Live demonstrations or ASMR content that pair sounds with craft narratives (e.g., fermenting sourdough) could help to enhance diners’ sensory engagement. Food practitioners might also leverage virtual classes with amplified food sounds to replicate workshop benefits globally [49]. Artisanal producers, in particular, can leverage the multisensory aspects of their craft, such as the unique sounds of stretching and folding dough or scoring bread, to create more immersive experiences for customers, whether in workshops, open kitchens, or digital media. By emphasizing the sensory richness and authenticity of artisanal methods, producers can differentiate their products and foster deeper consumer connections. In sum, this study positions sound as a dynamic, often underutilized tool in gastronomy—one that bridges science, tradition, and emotion. By nurturing audiovisual familiarity with food processes, chefs and researchers can transform passive listening into an immersive dialogue between plate and perception [50].

## 5. Conclusions

This study assessed how prior audiovisual experience with bread-making (hands-on workshop vs. watching food-making videos online) impacted people’s perception and preference for bread sounds. The results demonstrate that participants in bread-making workshops developed stronger associations with bread sounds, reported higher comfort levels, and experienced reduced annoyance compared to those who only watched videos. The findings underscore the critical role of prior sensory experiences in shaping individual preferences for food sounds. From a cognitive perspective, the effects of multisensory interventions fostering richer, more resonant food experiences indicate the importance of memory associated with prior experiences in determining the valence of sensory evaluation. Practically, the findings offer valuable guidance for culinary professionals, food marketers, and sensory scientists. Integrating sensory-rich workshops, dining experiences, and digital content can deepen consumer engagement and potentially enhance their preference for relevant auditory cues. However, the study’s multisensory design limits the explanation regarding which sensory modalities may be most relevant in constructing meaningful sensory memories, and conclusions may be limited to bread sounds. Regardless, this study serves as empirical evidence showing that sensory preferences are malleable.

## Figures and Tables

**Table 1:** Subjective rating dimensions and item scales.

Dimension	1 = Very Slightly or Not at All, 2 = A Little, 3 = Moderately, 4 = Quite a Bit, 5 = Extremely	
Comfort	To what extent do you agree or disagree with the following statements:	The sound is comforting.
Recognition		I can recognize the sound.
Annoyance		The sound is annoying.
Appetizingness		The sound makes me hungry.
Use of vision	Please indicate how much your senses contributed to your experience. “sight”	
Use of hearing	Please indicate how much your senses contributed to your experience. “sound”	

**Table 2:** Means (*M*s) and standard deviations (*SD*s) of soundtrack evaluations prior to and after the audiovisual association interventions (workshop vs. online).

Variable	Soundtrack	Workshop		Online	
		Pre *M* (*SD*)	Post *M* (*SD*)	Pre *M* (*SD*)	Post *M* (*SD*)
Recognition	Chewing	4.31 (1.15)	4.71 (0.82)	4.50 (0.80)	4.27 (1.19)
	Cutting	4.08 (1.27)	4.90 (0.31)	3.12 (1.42)	3.58 (1.32)
	Stroking	3.02 (1.51)	4.33 (0.95)	2.69 (1.29)	3.42 (1.19)
Comfort	Chewing	1.87 (0.94)	3.06 (1.24)	1.29 (0.57)	1.67 (1.02)
	Cutting	1.69 (1.19)	2.67 (1.31)	1.33 (0.76)	1.92 (1.04)
	Stroking	2.85 (1.20)	3.60 (1.14)	2.06 (1.16)	2.40 (1.14)
Annoyance	Chewing	2.90 (1.32)	2.31 (1.22)	4.00 (1.10)	3.52 (1.53)
	Cutting	2.98 (1.41)	2.65 (1.34)	3.63 (1.31)	3.19 (1.40)
	Stroking	1.75 (0.91)	1.48 (0.77)	2.58 (1.40)	2.23 (1.29)
Hunger/Appetite	Chewing	2.21 (1.22)	2.75 (1.10)	1.75 (0.99)	2.08 (1.27)
	Cutting	1.85 (1.07)	2.69 (1.15)	1.31 (0.61)	2.12 (1.21)
	Stroking	2.42 (1.38)	2.77 (1.29)	1.60 (1.03)	2.02 (1.20)

Note. Scales were from 1 to 5. “Pre” refers to the self-reported perception of the soundtracks before attending the workshop/watching videos. “Post” refers to the self-reported perception of the soundtracks after attending the workshop/watching videos.

**Table 3:** Means (*M*s) and standard deviations (*SD*s) of the intensities of vision and hearing engagement in each stage of the workshop vs. online video watching.

Variable	Procedure	Workshop *M* (*SD*)	Online *M* (*SD*)
Vision	Mixing	4.29 (0.90)	3.63 (0.82)
	Folding	4.35 (0.91)	3.56 (0.87)
	Bread Baked	4.15 (1.01)	4.04 (0.71)
Hearing	Mixing	2.63 (1.23)	3.88 (1.00)
	Folding	1.90 (1.06)	3.79 (0.87)
	Bread Baked	4.23 (0.97)	3.92 (0.88)

Note. Participants in the workshop group were also invited to experience bread tasting while participants online were only able to see the video of freshly baked bread. Scales were from 1 to 5.

**Table 4:** Effects of audiovisual association (workshop vs. online) and time (pre- and post- intervention) on sound perception (recognition, comfort, annoyance, and appetizingness).

Soundtrack	DV	Means (SE)				Interaction	Contrast Within Workshop	Contrast Within Online
		Pre		Post				
		Workshop	Online	Workshop	Online			
Chewing	Recognition	4.24 (0.15)	4.57 (0.14)	4.71 (0.16)	4.27 (0.15)	F(1,95) = 9.17 *p* = 0.003	F(1,95) = 7.39 *p* = 0.008	F(1,95) = 3.27 *p* = 0.074
	Comfort	1.85 (0.11)	1.31 (0.11)	3.04 (0.17)	1.69 (0.16)	F(1,95) = 12.75 *p* = 0.002	F(1,95) = 59.64 *p* < 0.001	F(1,95) = 6.73 *p* = 0.011
	Annoyance	2.96 (0.19)	3.94 (0.18)	2.42 (0.21)	3.42 (0.21)	F(1,95) = 0.01 *p* = 0.928	F(1,95) = 6.92 *p* = 0.010	F(1,95) = 6.83 *p* = 0.010
	Appetizingness	2.11 (0.17)	1.84 (0.16)	2.77 (0.19)	2.06 (0.18)	F(1,95) = 3.01 *p* = 0.086	F(1,95) = 14.25 *p* < 0.001	F(1,95) = 1.65 *p* = 0.202
Cutting	Recognition	4.04 (0.20)	3.15 (0.19)	4.86 (0.15)	3.61 (0.15)	F(1,95) = 1.61 *p* = 0.208	F(1,95) = 18.05 *p* < 0.001	F(1,95) = 6.23 *p* = 0.014
	Comfort	1.56 (0.14)	1.44 (0.13)	2.56 (0.18)	2.02 (0.17)	F(1,95) = 2.92 *p* = 0.091	F(1,95) = 34.76 *p* < 0.001	F(1,95) = 12.55 *p* < 0.011
	Annoyance	3.19 (0.20)	3.44 (0.19)	2.75 (0.21)	3.10 (0.20)	F(1,95) = 0.12 *p* = 0.730	F(1,95) = 4.94 *p* = 0.029	F(1,95) = 3.22 *p* = 0.076
	Appetizingness	1.80 (0.13)	1.36 (0.13)	2.75 (0.18)	2.06 (0.17)	F(1,95) = 1.05 *p* = 0.309	F(1,95) = 31.59 *p* < 0.001	F(1,95) = 18.57 *p* < 0.001
Stroking	Recognition	3.01 (0.21)	2.70 (0.20)	4.33 (0.17)	3.43 (0.16)	F(1,95) = 3.98 *p* = 0.049	F(1,95) = 41.76 *p* < 0.001	F(1,95) = 13.65 *p* < 0.001
	Comfort	2.77 (0.18)	2.13 (0.17)	3.65 (0.18)	2.36 (0.17)	F(1,95) = 7.45 *p* = 0.008	F(1,95) = 29.39 *p* < 0.001	F(1,95) = 2.19 *p* = 0.143
	Annoyance	1.84 (0.19)	2.49 (0.18)	1.51 (0.17)	2.21 (0.16)	F(1,95) = 0.05 *p* = 0.827	F(1,95) = 4.45 *p* = 0.038	F(1,95) = 3.50 *p* = 0.065
	Appetizingness	2.40 (0.18)	1.61 (0.17)	2.89 (0.18)	1.91 (0.17)	F(1,95) = 0.47 *p* = 0.496	F(1,95) = 6.55 *p* = 0.012	F(1,95) = 2.65 *p* = 0.107

Note. Each contrast within the between-group factor tests the multivariate simple effects of *time* within each level combination of the other effects shown (including covariates, i.e., age, sex, and baking experiences). These tests are based on the linearly independent pairwise comparisons among the estimated marginal means.

**Table 5:** Pearson correlations of the variables measured by self-reported scales in the study.

Variables	Comfort	Recognition	Annoyance	Appetizingness	Use of Vision	Use of Hearing
Comfort	1.00					
Recognition	0.38 ***	1.00				
Annoyance	−0.46 ***	−0.14	1.00			
Appetizingness	0.53 ***	0.31 **	−0.44 **	1.00		
Use of vision	0.23 *	0.15	−0.23	0.07	1.00	
Use of hearing	−0.13	−0.19	−0.010	−0.04	−0.09	1.00

Note. The variables’ values were averaged across sections (i.e., mixing, folding, and tasting). *** Correlation is significant at the 0.001 level (2-tailed). ** Correlation is significant at the 0.01 level (2-tailed). * Correlation is significant at the 0.05 level (2-tailed).

## Data Availability

The original contributions presented in this study are included in the article. Further inquiries can be directed to the corresponding authors.

## Appendix A

**Table A1:** A full list of the questionnaires used in the study.

Question/Item	Scale
Age	____ years
Sex	Male/Female/Prefer not to say
How much experience/expertise do you have in baking?	1 (“This is my first time”)
	2 (“I have a little bread making experiences”)
	3 (“I occasionally make a loaf at home”)
	4 (“I often make bread at home”)
	5 (“I am a professional baker”)
To what extent do you agree or disagree with the following statements:	1 (“Very slightly or not at all”)
The sound is comforting.	2 (“A little”)
I can recognize the sound.	3 (“Moderately”)
The sound is annoying.	4 (“Quite a bit”)
The sound makes me hungry.	5 (“Extremely”)
Indicate to what extent you feel this way right now, that is, at the present moment.	
Alert	1 (“Very slightly or not at all”) 2 (“A little”) 3 (“Moderately”) 4 (“Quite a bit”) 5 (“Extremely”)
Excited	
Nervous	
Attentive	
Enthusiastic	
Stressed	
Relaxed	
Connected to nature	
How much did you use each of your senses during this part of the experience.	
Sight	1 (“Very slightly or not at all”)
SAler	2 (“A little”)
Smell (workshop only)	3 (“Moderately”)
Touch (workshop only)	4 (“Quite a bit”)
Taste (workshop only)	5 (“Extremely”)
